# Alanine aminotransferase to high- density lipoprotein cholesterol ratio is positively correlated with the occurrence of diabetes in the Chinese population: a population-based cohort study

**DOI:** 10.3389/fendo.2023.1266692

**Published:** 2023-11-28

**Authors:** Shiming He, Changhui Yu, Maobin Kuang, Jiajun Qiu, Ruijuan Yang, Shuhua Zhang, Guotai Sheng, Yang Zou

**Affiliations:** ^1^ Department of Internal Medicine, Medical College of Nanchang University, Jiangxi Provincial People’s Hospital, Nanchang, Jiangxi, China; ^2^ Jiangxi Provincial Geriatric Hospital, Jiangxi Provincial People’s Hospital, The First Affiliated Hospital of Nanchang Medical College, Nanchang, Jiangxi, China; ^3^ Jiangxi Cardiovascular Research Institute, Jiangxi Provincial People’s Hospital, The First Affiliated Hospital of Nanchang Medical College, Nanchang, Jiangxi, China; ^4^ Department of Endocrinology, Jiangxi Provincial People’s Hospital, The First Affiliated Hospital of Nanchang Medical College, Nanchang, Jiangxi, China

**Keywords:** ALT/HDL-C ratio, diabetes, predictor, Chinese, cohort study

## Abstract

**Objective:**

Both alanine aminotransferase (ALT) and high-density lipoprotein cholesterol (HDL-C) are closely related to glucose homeostasis in the body, and the main objective of this study was to investigate the association between ALT to HDL-C ratio (ALT/HDL-C ratio) and the risk of diabetes in a Chinese population.

**Methods:**

The current study included 116,251 participants who underwent a healthy physical examination, and the study endpoint was defined as a diagnosis of new-onset diabetes. Multivariate Cox regression models and receiver operator characteristic curves were used to assess the association of the ALT/HDL-C ratio with diabetes onset.

**Results:**

During the average observation period of 3.10 years, a total of 2,674 (2.3%) participants were diagnosed with new-onset diabetes, including 1,883 (1.62%) males and 791 (0.68%) females. After fully adjusting for confounding factors, we found a significant positive association between the ALT/HDL-C ratio and the risk of diabetes [Hazard ratios 1.06, 95% confidence intervals: 1.05, 1.06], and this association was significantly higher in males, obese individuals [body mass index ≥ 28 kg/m^2^] and individuals aged < 60 years (All *P* interaction < 0.05). In addition, the ALT/HDL-C ratio was significantly better than its components ALT and HDL-C in predicting diabetes in the Chinese population.

**Conclusion:**

There was a positive relationship between ALT/HDL-C ratio and diabetes risk in the Chinese population, and this relationship was significantly stronger in males, obese individuals, and individuals younger than 60 years old.

## Introduction

Diabetes is a metabolic disorder characterized by high blood sugar levels and is one of the most common and rapidly growing diseases globally (1, 2). A recent systematic review, published in the Lancet, analyzed the diabetes burden across 204 countries. The findings revealed a staggering 90.5% surge in the global age-standardized diabetes prevalence from 1990 to 2021. In 2021 alone, the global diabetic population reached 529 million, and projections suggest that by 2050, this number will exceed 1.31 billion (3). In line with the global epidemic trend, the number of diabetic patients in China has increased rapidly in recent years. After age standardization, the number of diabetic patients in China accounted for 22.31% of the global total in 2021, and has become the center of the global diabetes pandemic (3). In addition to the high incidence, it is important to note that diabetes is associated with various complications, significantly increasing the risk of stroke and cardiovascular events (4–6), reducing the quality of life, and even posing a threat to life. Therefore, early detection and prevention are crucial in the management of diabetes.

High-density lipoprotein cholesterol (HDL-C), as a common marker of atherosclerosis (7), has been shown to have cardioprotective and anti-diabetic effects (8–10), and its levels and functions are related to glucose homeostasis (11), particularly in individuals with type 2 diabetes and hypertension (12–14). The liver, as a vital organ in the human body, plays a significant role in maintaining glucose homeostasis and insulin resistance (IR) (15). Alanine aminotransferase (ALT) is one of the most important indicators reflecting liver function (16). It primarily converts alanine to pyruvate in the process of liver glucose regulation to produce glucose, playing a crucial role in gluconeogenesis and is closely related to IR and the progression of diabetes (17–19). Recently, researchers have investigated the combination of ALT and HDL-C, finding that the ratio of ALT to HDL-C (ALT/HDL-C ratio) is a useful new predictor for the risk of developing diabetes in the Japanese population (20). However, it remains unclear whether there is an association between the ALT/HDL-C ratio and diabetes in the Chinese population. To address this question, the current study conducted a new analysis based on national health examination data from the China Rich Medical Group.

## Methods

### Data source and study design

**Figure 1:**
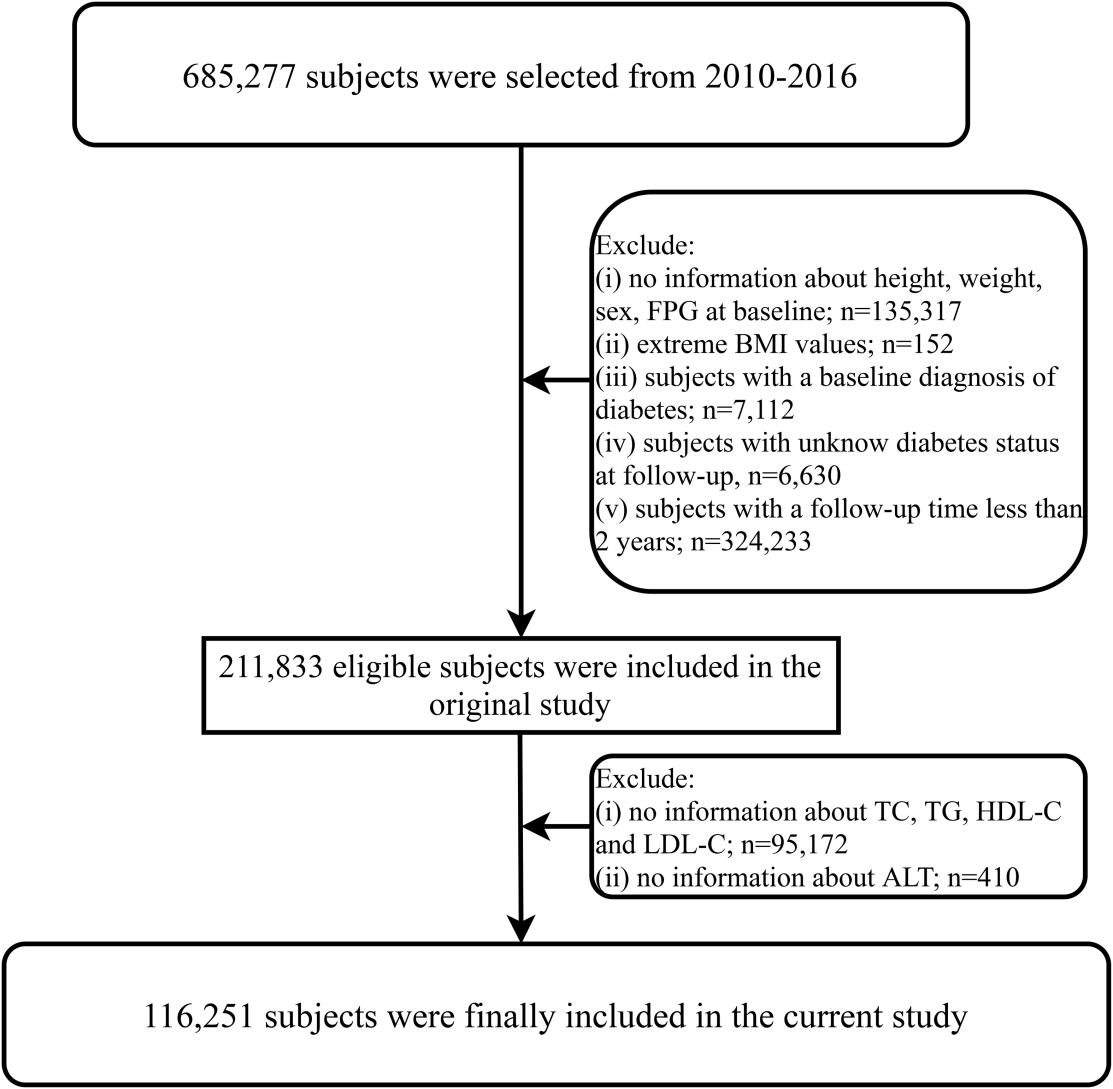
Flow chart for inclusion and exclusion of study participants. FPG, fasting plasma glucose; BMI, body mass index; TG, triglycerides; TC, total cholesterol; LDL-C, low-density lipoprotein cholesterol; HDL-C, high-density lipoprotein cholesterol; ALT, alanine aminotransferase.

The current study is a secondary analysis of a large longitudinal cohort study conducted in China. Briefly, the longitudinal study aimed to assess chronic diseases and their risk factors. The original cohort consisted of 685,277 participants who underwent health examinations at the Rich Healthcare Group in 11 cities (Nantong, Wuhan, Hefei, Guangzhou, Chengdu, Changzhou, Shenzhen, Suzhou, Nanjing, Beijing, Shanghai) in China between 2010 and 2016. These participants underwent various measurements, including general physical parameter assessments, lifestyle evaluations, and blood tests as part of the medical examinations. The available data from the longitudinal cohort have been uploaded to the Dryad database for sharing by Chen et al. (21), and we utilized these data for our secondary analysis while respecting the authors’ rights. In a previous study, Chen et al. analyzed the relationship between body mass index (BMI) and diabetes, and the detailed research design and methods have been published elsewhere (22), in which subjects with the following characteristics were excluded from their study: (1) diagnosed with diabetes at baseline, (2) unknown glycemic status during follow-up, (3) follow-up duration less than 2 years, (4) missing or extreme values of sex, height, weight, BMI, and fasting plasma glucose (FPG), and (6) participants who withdrew from the study for unknown reasons. Ultimately, they included 211,833 participants for analysis (22). In the current study, we extracted the clinical data uploaded by Chen et al. from the Dryad database and further excluded participants with missing baseline lipid parameters (n=95,172) and ALT parameter data (n=410) based on the research objectives. Finally, we included 116,251 participants. **Figure 1** illustrates the flowchart of this study. The current study is a secondary analysis, and the new research protocol has been authorized by the ethics committee of the authors’ institution (Jiangxi Provincial People’s Hospital Ethical Approval Number: 2021-067), which oversaw the entire research process to ensure compliance with the Helsinki Declaration. Additionally, the Ethics Committee of Jiangxi Provincial People’s Hospital waived the requirement to obtain informed consent, given that the current study data did not contain subject’ identity information.

### Health examinations and laboratory measurements

All baseline data included in the database were collected through standardized questionnaires during the initial assessment, which encompassed age, sex, height, weight, systolic blood pressure (SBP), diastolic blood pressure (DBP), family history of diabetes, smoking, and drinking status. General physical parameters were measured by trained medical personnel in a quiet indoor environment, with weight measured to an accuracy of 0.1 kilograms and height measured to an accuracy of 0.1 centimeters.

Prior to each participant’s visit, they were required to fast for at least 10 hours. Venous blood samples were collected at the examination center, and professional healthcare personnel measured the levels of serum triglycerides (TG), total cholesterol (TC), low-density lipoprotein cholesterol (LDL-C), HDL-C, FPG, ALT, aspartate transaminase (AST), blood urea nitrogen (BUN), and creatinine (Cr) using the Beckman 5800 Automatic Biochemical Analyzer

### Definitions and calculations

The start of follow-up was defined as the time of the initial health assessment, and the endpoint of follow-up was the diagnosis of incident diabetes or the end of the study (whichever occurred first). According to the diagnostic criteria of the American Diabetes Association, diabetes was defined as an FPG measurement ≥ 7.00 mmol/L during the follow-up period or self-reported diagnosis of diabetes (23).

Smoking status: At baseline data collection, participants were categorized into four groups based on their smoking history: non-smoker, former smoker, current smoker, and not recorded.

Drinking status: At baseline data collection, participants were categorized into four groups based on their alcohol consumption history: non-drinker, former drinker, current drinker, and not recorded.

BMI was calculated as weight divided by height squared, and participants were classified according to the BMI categories for the Chinese population: < 24 kg/m^2^ (non-obese), ≥ 24 kg/m^2^, and < 28 kg/m^2^ (overweight), ≥ 28 kg/m^2^ (obese) (24).

### Statistical analysis

Data analysis was performed using R software version 3.4.3 and Empower(R) software version 2.20. A two-sided *P*-value < 0.05 was considered statistically significant.

Baseline characteristics were compared among groups defined by quintiles of the ALT/HDL-C ratio, including proportions of study participants, means, or medians. Differences in continuous variables among the groups were compared using one-way analysis of variance or the Kruskal-Wallis H test, while differences in categorical variables were compared using the chi-square test. Cumulative incidence curves for the primary endpoint in each quintile of the ALT/HDL-C ratio were estimated using the Kaplan-Meier method, and differences between groups were compared using the log-rank test.

To further explore the relationship between the ALT/HDL-C ratio and diabetes risk, the ALT/HDL-C ratio was included in Cox regression models both as a continuous variable and a categorical variable, and Hazard ratios (HRs) with 95% confidence intervals (CI) were recorded. Collinearity among covariates was assessed using multiple linear regression analysis, with the variance inflation factor used as an assessment tool for collinearity between covariates (**Supplementary Table 1**) (25). Initially, an unadjusted Crude Model was evaluated and served as the reference for subsequent models. Model 1 was adjusted for sex, BMI, and age based on the Crude Model; to account for potential influences of family history and lifestyle habits factors on diabetes risk, they were further adjusted in Model 2. Finally, to assess the independent relationship between the ALT/HDL-C ratio and diabetes, potential influences of blood pressure (SBP, DBP), blood glucose (FPG), blood lipids (TG, LDL-C), BUN, and renal function (Cr) were further considered in Model 3. Furthermore, to mitigate the potential influence of undiagnosed liver diseases, we continued to exclude participants with abnormal liver function (baseline ALT or AST higher than 40 IU/L) in the current study and followed the aforementioned analytical procedures.

The predictive performance of the ALT/HDL-C ratio and its components (ALT and HDL-C) for diabetes was assessed using receiver operator characteristic (ROC) curves, and the corresponding area under the curves (AUCs) was calculated. Their performance was compared using the Delong test. Finally, considering the important influence of factors such as age, sex, BMI, and family history of diabetes on the risk of diabetes, we further performed subgroup analyses based on these factors in Model 3, and examined the differences between groups by likelihood ratio test.

## Results

### Baseline characteristics

After excluding participants who did not meet the criteria, a total of 116,251 baseline normoglycemic participants were included, with an average age of 44 years, including 62,622 males and 53,629 females. **Table 1** presents the baseline characteristics of participants stratified by quintiles of the ALT/HDL-C ratio. From the table, it can be observed that as the quintiles of the ALT/HDL-C ratio increase, the proportion of male participants gradually increased, while the proportion of female participants gradually decreased. In addition, we found that except for HDL-C, which showed a decreasing trend, other indicators such as height, weight, BMI, SBP, DBP, FPG, TC, TG, LDL-C, ALT, AST, BUN, and Cr levels increased with increasing quintiles of the ALT/HDL-C ratio.

**Table 1:** Baseline characteristics of participants grouped according to ALT/HDL-C ratio quintiles.

	ALT/HDL-C ratio quintiles					*P*-value
	Q1 (<8.28)	Q2 (8.28-11.57)	Q3 (11.57-16.18)	Q4 (16.19-24.92)	Q5 (>24.92)	
No. of participants	23240	23256	23253	23251	23251	
Age (years)	37.00 (32.00-47.00)	41.00 (34.00-53.00)	44.00 (35.00-56.00)	44.00 (35.00-56.00)	41.00 (34.00-51.00)	<0.001
Sex						<0.001
Male	3959 (17.04%)	8487 (36.49%)	13207 (56.80%)	17102 (73.55%)	19867 (85.45%)	
Female	19281 (82.96%)	14769 (63.51%)	10046 (43.20%)	6149 (26.45%)	3384 (14.55%)	
Height (cm)	162.48 (7.05)	164.21 (8.06)	166.28 (8.44)	168.38 (8.07)	170.15 (7.53)	<0.001
Weight (kg)	55.93 (8.06)	60.07 (9.45)	64.52 (10.35)	69.15 (10.72)	74.78 (11.72)	<0.001
BMI (kg/m^2^)	21.15 (2.51)	22.23 (2.77)	23.28 (2.93)	24.33 (2.96)	25.76 (3.19)	<0.001
SBP (mmHg)	112.87 (15.27)	117.08 (16.65)	120.46 (16.84)	122.55 (16.41)	124.20 (15.64)	<0.001
DBP (mmHg)	70.23 (9.85)	72.54 (10.52)	74.62 (10.83)	76.50 (10.90)	78.31 (10.85)	<0.001
FPG (mmol/L)	4.82 (0.54)	4.90 (0.57)	4.96 (0.60)	5.01 (0.63)	5.05 (0.66)	<0.001
TC (mmol/L)	4.72 (0.85)	4.71 (0.89)	4.76 (0.90)	4.83 (0.90)	4.93 (0.92)	<0.001
TG (mmol/L)	0.78 (0.60-1.04)	0.92 (0.69-1.30)	1.10 (0.80-1.56)	1.33 (0.95-1.92)	1.70 (1.20-2.46)	<0.001
HDL-C(mmol/L)	1.62 (0.30)	1.45 (0.25)	1.36 (0.25)	1.27 (0.25)	1.17 (0.26)	<0.001
LDL-C (mmol/L)	2.60 (2.23-3.03)	2.64 (2.24-3.09)	2.70 (2.29-3.17)	2.75 (2.34-3.22)	2.82 (2.39-3.30)	<0.001
ALT (IU/L)	10.30 (9.00-12.00)	14.00 (12.30-16.00)	18.00 (16.00-21.00)	24.80 (21.20-28.80)	41.90 (33.60-56.30)	<0.001
AST (IU/L)	18.00 (15.90-20.40)	20.00 (17.30-22.60)	22.00 (19.00-25.00)	24.00 (21.00-27.60)	30.40 (25.90-37.80)	<0.001
BUN (mmol/L)	4.24 (3.57-5.05)	4.47 (3.75-5.30)	4.65 (3.92-5.47)	4.72 (4.01-5.55)	4.72 (4.02-5.50)	<0.001
Cr (mmol/L)	59.50 (53.10-68.20)	64.00 (55.20-77.00)	71.00 (59.00-82.30)	75.30 (64.30-84.90)	77.00 (68.00-85.50)	<0.001
Family history of diabetes						0.041
No	22698 (97.67%)	22699 (97.60%)	22769 (97.92%)	22761 (97.89%)	22696 (97.61%)	
Yes	542 (2.33%)	557 (2.40%)	484 (2.08%)	490 (2.11%)	555 (2.39%)	
Smoking status						<0.001
Non	439 (1.89%)	808 (3.47%)	1313 (5.65%)	1785 (7.68%)	2301 (9.90%)	
Former	69 (0.30%)	179 (0.77%)	250 (1.08%)	342 (1.47%)	480 (2.06%)	
Current	4879 (20.99%)	4810 (20.68%)	4915 (21.14%)	4994 (21.48%)	5009 (21.54%)	
Not recorded	17853 (76.82%)	17459 (75.07%)	16775 (72.14%)	16130 (69.37%)	15461 (66.50%)	
Drinking status						<0.001
Non	78 (0.34%)	108 (0.46%)	177 (0.76%)	244 (1.05%)	265 (1.14%)	
Former	424 (1.82%)	736 (3.16%)	1180 (5.07%)	1403 (6.03%)	1765 (7.59%)	
Current	4885 (21.02%)	4953 (21.30%)	5121 (22.02%)	5474 (23.54%)	5760 (24.77%)	
Not recorded	17853 (76.82%)	17459 (75.07%)	16775 (72.14%)	16130 (69.37%)	15461 (66.50%)	

Values were expressed as mean (standard deviation) or medians (quartile interval) or n (%). BMI, body mass index; SBP, systolic blood pressure; DBP, diastolic blood pressure; FPG fasting plasma glucose; TG, triglyceride; TC, total cholesterol; LDL-C, low-density lipid cholesterol; BUN, blood urea nitrogen; Cr, creatinine; ALT, alanine aminotransferase; AST, aspartate aminotransferase; ALT/HDL-C ratio, alanine aminotransferase to high-density lipoprotein cholesterol ratio.

### Incidence of diabetes

**Figure 2:**
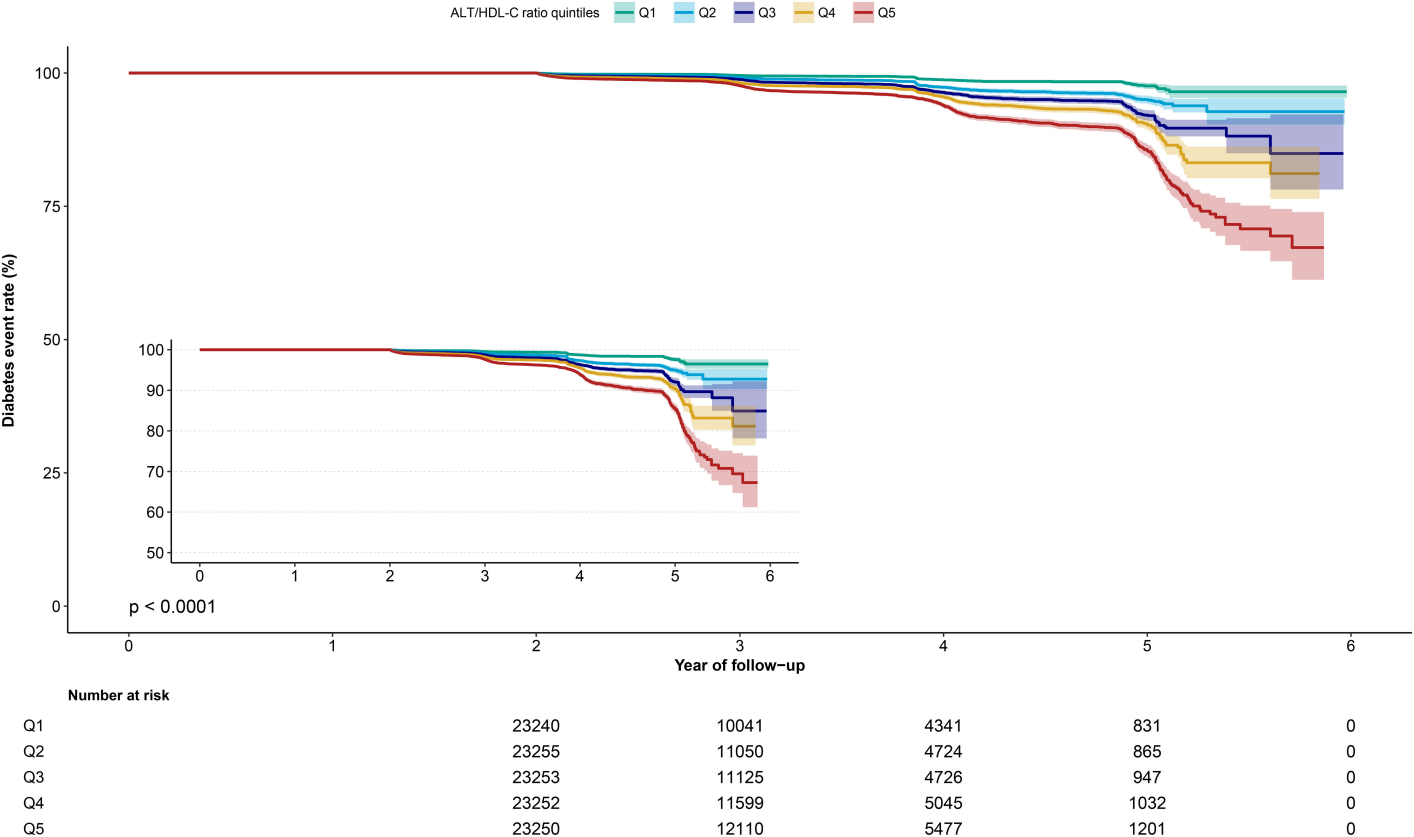
Kaplan-Meier curve of ALT/HDL-C ratio quintiles over time. ALT/HDL-C ratio, alanine aminotransferase to high-density lipoprotein cholesterol ratio.

During a median follow-up period of 3.10 ± 0.95 years, a total of 2,674 (2.3%) participants were diagnosed with incident diabetes. **Figure 2** displays the Kaplan-Meier curves of cumulative incidence of diabetes according to quintiles of the ALT/HDL-C ratio, showing an increasing trend in cumulative incidence of diabetes with increasing quintiles of the ALT/HDL-C ratio (log-rank *P* < 0.001).

### Association between ALT/HDL-C ratio and diabetes risk

**Table 2** summarizes the relationship between the ALT/HDL-C ratio and diabetes incidence in the multivariable Cox regression analysis. Four multivariable-adjusted models were established, and a positive trend was observed between the ALT/HDL-C ratio as a continuous variable and the risk of incident diabetes in the crude model and Models 1-3. When the ALT/HDL-C ratio was treated as a categorical variable, compared to the lowest quintile (Q1), the highest risk was consistently observed in the fifth quintile (Q5) in all models (*P*-trend < 0.0001). Although the HR values slightly decreased in the adjusted Models 1, 2, and 3 compared to the crude model, the overall positive trend remained unchanged.

**Table 2:** Cox regression analyses for the association between the ALT/HDL-C ratio and the incidence of diabetes.

	Hazard ratios (95% confidence interval)			
	Crude model	Model 1	Model 2	Model 3
ALT/HDL-C ratio (Per SD increase)	1.06 (1.05, 1.06)	1.06 (1.05, 1.07)	1.06 (1.05, 1.07)	1.06 (1.05, 1.08)
ALT/HDL-C ratio (Quintile)				
Quintile 1	Ref	Ref	Ref	Ref
Quintile 2	2.07 (1.70, 2.51)	1.35 (1.11, 1.64)	1.35 (1.11, 1.64)	1.23 (1.01, 1.51)
Quintile 3	3.08 (2.56, 3.70)	1.52 (1.26, 1.84)	1.54 (1.27, 1.86)	1.20 (0.99, 1.46)
Quintile 4	4.05 (3.39, 4.83)	1.72 (1.43, 2.07)	1.73 (1.44, 2.09)	1.18 (0.97, 1.43)
Quintile 5	5.87 (4.94, 6.97)	2.55 (2.12, 3.07)	2.56 (2.12, 3.08)	1.46 (1.20, 1.77)
*P*-trend	<0.0001	<0.0001	<0.0001	<0.0001

ALT/HDL-C ratio, alanine aminotransferase to high-density lipoprotein cholesterol ratio; SD, standard deviation.

Model 1 adjusted for age, sex height and BMI;

Model 2 adjusted for age, sex height, BMI, family history of diabetes, smoking status and drinking status;

Model 3 adjusted for age, sex height, BMI, family history of diabetes, smoking status, drinking status, SBP, DBP, FPG, TG, LDL-C, BUN and Cr.

As a sensitivity analysis, we further examined the association among participants with normal liver function. After excluding 13,079 participants with ALT or AST levels higher than 40 IU/L, we conducted the same analytical procedures on the remaining 103,172 participants, and the new analysis results aligned with the results of the whole population analysis, indicating a positive correlation between the ALT/HDL-C ratio and diabetes risk, with the correlation progressively intensifying across the ALT/HDL-C ratio quintiles (**Table 3**).

**Table 3:** Assessing the association between ALT/HDL-C ratio and incidence of diabetes in participants with normal liver function.

	No. of participants	Hazard ratios (95% confidence interval)			
		Crude model	Model 1	Model 2	Model 3
ALT/HDL-C ratio (Per SD increase)	103,172	1.34 (1.30, 1.38)	1.15 (1.10, 1.20)	1.15 (1.10, 1.20)	1.09 (1.04, 1.13)
ALT/HDL-C ratio (Quintile)					
Quintile 1	20631	Ref	Ref	Ref	Ref
Quintile 2	20588	1.84 (1.49, 2.28)	1.23 (0.99, 1.52)	1.23 (0.99, 1.52)	1.18 (0.95, 1.47)
Quintile 3	20672	2.81 (2.30, 3.43)	1.45 (1.18, 1.77)	1.46 (1.19, 1.79)	1.39 (1.13, 1.71)
Quintile 4	20644	3.96 (3.27, 4.80)	1.71 (1.40, 2.08)	1.73 (1.41, 2.11)	1.53 (1.25, 1.88)
Quintile 5	20637	4.38 (3.62, 5.29)	1.80 (1.47, 2.21)	1.82 (1.49, 2.23)	1.53 (1.24, 1.88)
*P*-trend		<0.0001	<0.0001	<0.0001	<0.0001

ALT/HDL-C ratio, alanine aminotransferase to high-density lipoprotein cholesterol ratio; SD, standard deviation.

Model 1 adjusted for age, sex height and BMI;

Model 2 adjusted for age, sex height, BMI, family history of diabetes, smoking status and drinking status;

Model 3 adjusted for age, sex height, BMI, family history of diabetes, smoking status, drinking status, SBP, DBP, FPG, TG, LDL-C, BUN and Cr.

### Subgroup analysis

Subgroup analysis was conducted based on age, sex, family history of diabetes, and BMI to explore the relationship between the ALT/HDL-C ratio and diabetes risk in different commonly categorized populations. The study revealed significant statistical differences (*P*-interaction < 0.05) in the association between the ALT/HDL-C ratio and diabetes risk among different age groups, sexes, and BMI groups. **Table 4** demonstrates that the risk of diabetes incidence varied among different age groups, with a higher risk observed in participants aged ≤ 60 years compared to those aged > 60 years (*P*-interaction < 0.0001). In the sex subgroup analysis, males had a higher risk of developing diabetes (HR: 1.08 for males, 1.04 for females; *P*-interaction = 0.0204). In the BMI subgroup analysis, compared to non-obese and overweight individuals, the highest diabetes risk related to the ALT/HDL-C ratio was observed in obese individuals (HR: non-obese 1.05 VS overweight 1.06 VS obese 1.17; *P*-interaction = 0.0001). Additionally, there was no statistically significant difference in the influence of family history of diabetes on the relationship between the ALT/HDL-C ratio and diabetes risk (*P* = 0.8113).

**Table 4:** Stratified association between the ALT/HDL-C ratio and diabetes by age, sex, BMI and family history of diabetes.

Subgroup	No. of participants	adjusted HR (95%CI)	*P* for interaction
Age (years)			<0.0001
20-30	11121	1.24 (1.14, 1.33)	
31-40	41806	1.24 (1.18, 1.30)	
41-50	27034	1.09 (1.07, 1.10)	
51-60	19436	1.03 (1.00, 1.07)	
61-70	11969	0.87 (0.79, 0.97)	
>70	4885	0.70 (0.57, 0.85)	
Sex			0.0204
Male	61472	1.08 (1.06, 1.10)	
Female	52124	1.04 (1.01, 1.08)	
Family history of diabetes			0.8113
Yes	2586	1.08 (0.94, 1.24)	
No	111010	1.06 (1.05, 1.08)	
BMI (kg/m^2^)			0.0001
<24	67611	1.05 (1.03, 1.07)	
≥24, <28	36102	1.06 (1.02, 1.10)	
≥28	9883	1.17 (1.12, 1.22)	

ALT/HDL-C ratio, alanine aminotransferase to high-density lipoprotein cholesterol ratio; HR, hazard ratios; CI, confidence interval; BMI, body mass index.

Models adjusted for the same covariates as in model 3 (**Table 2**), except for the stratification variable.

### Evaluation of ALT/HDL-C ratio for predicting diabetes

**Figure 3:**
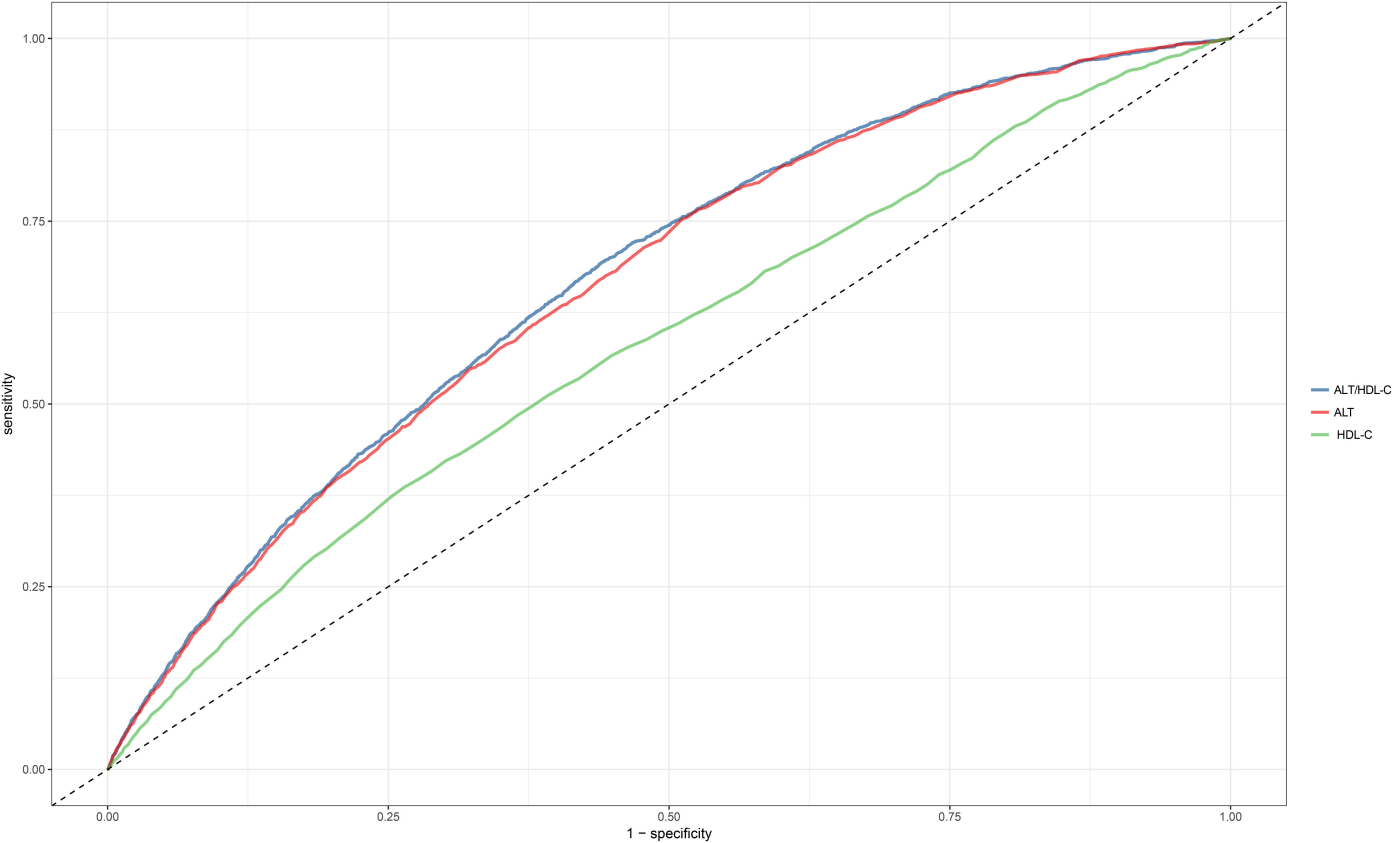
The ROC curve of the predicting efficiency of ALT, HDL-C and ALT/HDL-C ratio. ROC, receiver operator characteristic; AUC, area under the curve; ALT, alanine aminotransferase; HDL-C, high-density lipoprotein cholesterol; ALT/HDL-C ratio, alanine aminotransferase to high-density lipoprotein cholesterol ratio.

To compare the predictive value of the ALT/HDL-C ratio and its components for incident diabetes, ROC curve analysis was performed, and the corresponding AUC values were calculated (**Figure 3**). The results indicated that the ALT/HDL-C ratio had the highest predictive value for incident diabetes compared to ALT and HDL-C alone (AUC: ALT/HDL-C ratio: 0.6716, ALT: 0.6653, HDL-C: 0.5817; all Delong *P* < 0.05). The optimal threshold point for the ALT/HDL-C ratio was calculated as 14.9248 (**Table 5**).

**Table 5:** Areas under the receiver operating characteristic curves for each evaluated parameter in identifying diabetes.

	AUC	95%CI low	95%CI up	Best threshold	Specificity	Sensitivity
ALT*	0.6653	0.6555	0.6751	17.9500	0.4887	0.7524
HDL-C*	0.5817	0.5707	0.5928	1.1750	0.7359	0.3859
ALT/HDL-C ratio	0.6716	0.6618	0.6813	14.9248	0.5600	0.6937

AUC, area under the curve; ALT, alanine aminotransferase; HDL-C, high-density lipoprotein cholesterol; ALT/HDL-C ratio, alanine aminotransferase to high-density lipoprotein cholesterol ratio. *P<0.05, compare with ALT/HDL-C ratio (Delong test).

## Discussion

This study revealed, for the first time, a positive association between the ALT/HDL-C ratio and the risk of diabetes in the Chinese population. Additionally, ROC curve analysis showed that the ALT/HDL-C ratio had better predictive performance for diabetes compared to ALT and HDL-C alone. Subgroup analyses based on age, sex, family history of diabetes, and BMI demonstrated significant differences in the association between the ALT/HDL-C ratio and diabetes risk among different age groups, sexes, and BMI groups, but not among diabetes family history groups.

ALT is an important indicator reflecting liver function (16). In recent years, numerous studies have shown a strong correlation between elevated serum ALT levels and metabolic dysfunction, particularly in glucose metabolism (26–28). It has been found that ALT is positively associated with the risk of diabetes in both young and middle-aged to older populations (29–35). However, the mechanisms underlying the association between ALT and diabetes risk remained unclear. Some researchers suggested that the increased risk of diabetes associated with elevated ALT levels may be related to decreased liver insulin sensitivity (36), while others proposed that the elevated ALT levels may be a consequence of IR in participants, and ALT, in turn, increased the risk of diabetes (37). HDL is a common lipoprotein that plays a protective role in heart and vascular function through reverse cholesterol transport (38, 39). In recent years, increasing evidence has shown that HDL not only influences cardiovascular diseases but also plays a significant role in the development of diabetes (13, 40). The mechanisms by which HDL influences diabetes are believed to be related to HDL function and its major apolipoprotein, apoA-I (41–43). They regulate glucose metabolism by improving insulin sensitivity and increasing insulin secretion (44).

The ALT/HDL-C ratio is a new combination index recently studied, which is first proposed by Cao et al. (20). In terms of its constituent variables, this parameter takes into account the influence of liver function and atherosclerosis; considering that ALT and HDL-C are closely related to blood glucose metabolism (13, 29–36, 40), the combination of the two might provide a more effective assessment/prediction of diabetes incidents. This hypothesis was verified in a subsequent study conducted by Cao et al. based on the Japanese population (20) and in our study based on the Chinese population. In both studies, we found a positive association between ALT/HDL-C ratio and diabetes, and the ALT/HDL-C ratio proved to be a more potent predictor of diabetes than either ALT or HDL-C alone. Compared with the study conducted by Cao et al., the current study had significant differences in the study population; in addition, the optimal threshold of ALT/HDL-C ratio for diabetes prediction should also be noted: compared with the Japanese population, the current study calculated that the optimal threshold for predicting diabetes is relatively lower (14.9248 vs 17.56), which also suggests that the Chinese population may need more stringent standards in future diabetes risk assessment/prediction.

In the current study, a more detailed subgroup analysis revealed intriguing findings: specifically, the association between ALT/HDL-C ratio and diabetes was notably stronger in males, overweight/obese individuals, and individuals younger than 60 years old. The cause of this particular phenomenon is believed to be related to the ALT and HDL-C levels corresponding to these subgroups. The main analysis is as follows: (1) Males and overweight/obese people generally have higher ALT levels (45, 46) and lower HDL-C levels than females and non-obese people (47–50); from a numerical analysis, higher ALT or lower HDL-C means that the ALT/HDL-C ratio will increase; Judging from the results, an elevated ALT/HDL-C ratio corresponded to a heightened risk of diabetes in males and overweight/obese people. (2) From the longitudinal relationship between ALT, HDL-C and age, ALT usually decreases gradually with age (51, 52), while HDL-C usually changes less (48, 53, 54); these characteristics will further lead to a reduction in ALT/HDL-C ratio values, thereby reducing the risk of diabetes related to ALT/HDL-C ratio in people over 60 years old. In addition, it should be noted that older people generally have larger HDL particle size, a phenotype that is closely associated with a lower risk of diabetes (55).

As a new index, the specific mechanism by which the ALT/HDL-C ratio leads to diabetes remains unclear. However, numerous studies have shown that ALT and HDL are both associated with IR (36, 44), suggesting that the mechanism by which the ALT/HDL-C ratio leads to diabetes may be closely related to IR. As we know, IR, as a pathogenic driver of metabolic diseases, is defined as reduced sensitivity of target organs to the action of insulin (56). It has been suggested that the mechanisms leading to IR are primarily two arguments, lipid overload and inflammation (57), which are roughly the same as the mechanisms by which ALT and HDL act alone. Therefore, we believed that the ALT/HDL-C ratio may affect the development of diabetes by mediating IR. In the future, quantifying IR levels may help validate the specific mechanism of the association between the ALT/HDL-C ratio and diabetes.

Up to now, there are few studies on the relationship between ALT/HDL-C ratio and diabetes. However, from the current research results and published results (20), the ALT/HDL-C ratio is a useful parameter for evaluating the risk of diabetes in the Chinese population and Japanese population, and it is superior to ALT and HDL-C alone. The consistency of these findings may provide valuable insights for future research or strategies in predicting diabetes, some of which are summarized below: (1) Offering reference materials for subsequent related research in other ethnic groups. (2) Introducing another simple and usable strategy for diabetes risk assessment. (3) Inspiring fresh perspectives in constructing or enhancing diabetes risk prediction models. (4) Highlighting the relationship between the ALT/HDL-C ratio and diabetes can enhance public awareness of diabetes, emphasizing that diabetes isn’t solely about blood sugar fluctuations but also impacts multiple systems within the body. Based on the above considerations, for future suggestions or research directions, we believe that both the general public, medical staff and researchers should pay attention to the changes of other metabolic factors besides hyperglycemia in their cognition of diabetes, which also has an important impact on the prevention of diabetes.

### Study strengths and limitations

Strengths: (1) This study involved a large sample size of 116,251 participants, which makes the results of the current study relatively reliable. (2) The current study revealed, for the first time, a positive correlation between the ALT/HDL-C ratio and diabetes risk in the Chinese population. (3) Several subgroup analyses were performed to compare differences in the correlation between ALT/HDL-C ratio and diabetes risk in populations with different characteristics, and these findings inform the clinical application of the ALT/HDL-C ratio.

Limitations: (1) Although the current research population hailed from various cities across China, the majority (10 out of 11 cities) are situated in the southern region. Therefore, the evidence of the current research may be more suitable for the population in the south of China, and the relevance of these results to the northern Chinese demographic requires further investigation. (2) The current study dataset is sourced from a public database and lacks liver disease information, detailed alcohol consumption information, and drug use information. considering that these factors may affect liver function, subsequently impacting the ALT/HDL-C ratio and the study’s outcomes, the absence of these information introduce certain limitations to our research. However, as an alternative solution, we further excluded the subjects whose baseline ALT and AST exceeded the normal reference level and conducted the same analysis steps; the results showed that the relationship between ALT/HDL-C ratio and diabetes remained positive in the population with normal liver function. This result largely reduces the potential impact of changes in liver function due to other causes and supports the conclusions of the main analysis. (3) Only baseline measurements of ALT, HDL-C, and other parameter indicators were considered, without accounting for numerical variations in ALT/HDL-C ratio over time. (4) The study did not evaluate data on postprandial blood glucose levels, which may have resulted in the underdiagnosis of some diabetes cases. (5) The current study excluded a large number of people who did not meet the inclusion criteria, which would bring about a certain selection bias.

## Conclusion

In conclusion, the findings of this study conducted on the Chinese population demonstrated a positive correlation between the ALT/HDL-C ratio and the risk of developing diabetes. This association was particularly pronounced in males, individuals with obesity, and those aged ≤ 60 years. Moreover, these findings underscored the predictive value of the ALT/HDL-C ratio in assessing the risk of diabetes, providing valuable insights for early prevention and treatment of the disease.

## Data availability statement

The original contributions presented in the study are included in the article/**Supplementary Material**, further inquiries can be directed to the corresponding author/s.

## Ethics statement

The studies involving humans were approved by the ethics committee of Jiangxi Provincial People’s Hospital. The studies were conducted in accordance with the local legislation and institutional requirements. The ethics committee/institutional review board waived the requirement of written informed consent for participation from the participants or the participants’ legal guardians/next of kin because The Ethics Committee of Jiangxi Provincial People’s Hospital waived the requirement to obtain informed consent, given that the current study data did not contain subject’ identity information.

## Author contributions

SH: Data curation, Formal analysis, Software, Validation, Writing – original draft. CY: Data curation, Formal analysis, Software, Validation, Writing – original draft. MK: Conceptualization, Data curation, Formal analysis, Software, Validation, Writing – original draft. JQ: Writing – review & editing. RY: Formal analysis, Validation, Writing – review & editing. SZ: Writing – review & editing. GS: Conceptualization, Supervision, Writing – review & editing, Project administration. YZ: Conceptualization, Supervision, Writing – review & editing, Methodology.

## References

[1] Antar SA Ashour NA Sharaky M Khattab M Ashour NA Zaid RT. Diabetes mellitus: Classification, mediators, and complications; A gate to identify potential targets for the development of new effective treatments. BioMed Pharmacother (2023) 168:115734. doi: 10.1016/j.biopha.2023.115734 37857245

[2] Chen J Huang Y Liu C Chi J Wang Y Xu L. The role of C-peptide in diabetes and its complications: an updated review. Front Endocrinol (Lausanne) (2023) 14:1256093. doi: 10.3389/fendo.2023.1256093 37745697 PMC10512826

[3] GBD 2021 Diabetes Collaborators. Global, regional, and national burden of diabetes from 1990 to 2021, with projections of prevalence to 2050: a systematic analysis for the Global Burden of Disease Study 2021. Lancet (2023) 402(10397):203–34. doi: 10.1016/S0140-6736(23)01301-6 PMC10364581 37356446

[4] Elafros MA Andersen H Bennett DL Savelieff MG Viswanathan V Callaghan BC. Towards prevention of diabetic peripheral neuropathy: clinical presentation, pathogenesis, and new treatments. Lancet Neurol (2022) 21(10):922–36. doi: 10.1016/S1474-4422(22)00188-0 PMC10112836 36115364

[5] Mosenzon O Cheng AY Rabinstein AA Sacco S. Diabetes and stroke: what are the connections? J Stroke (2023) 25(1):26–38. doi: 10.5853/jos.2022.02306 36592968 PMC9911852

[6] Pop-Busui R Januzzi JL Bruemmer D Butalia S Green JB Horton WB. Heart failure: an underappreciated complication of diabetes. A Consensus Rep Am Diabetes Assoc Diabetes Care (2022) 45(7):1670–90. doi: 10.2337/dci22-0014 PMC9726978 35796765

[7] Rosenson RS Brewer HB Jr Ansell BJ Barter P Chapman MJ Heinecke JW. Dysfunctional HDL and atherosclerotic cardiovascular disease. Nat Rev Cardiol (2016) 13(1):48–60. doi: 10.1038/nrcardio.2015.124 26323267 PMC6245940

[8] Rotllan N Julve J Escolà-Gil JC. Type 2 Diabetes and HDL dysfunction: a key contributor to Glycemic Control. Curr Med Chem (2023) 31(3):280–5. doi: 10.2174/0929867330666230201124125 36722477

[9] Cochran BJ Manandhar B Rye KA. HDL and diabetes. Adv Exp Med Biol (2022) 1377:119–27. doi: 10.1007/978-981-19-1592-5_9 35575925

[10] Rohatgi A Westerterp M von Eckardstein A Remaley A Rye KA. HDL in the 21st century: A multifunctional roadmap for future HDL research. Circulation (2021) 143(23):2293–309. doi: 10.1161/CIRCULATIONAHA.120.044221 PMC8189312 34097448

[11] Xepapadaki E Nikdima I Sagiadinou EC Zvintzou E Kypreos KE. HDL and type 2 diabetes: the chicken or the egg? Diabetologia (2021) 64(9):1917–26. doi: 10.1007/s00125-021-05509-0 34255113

[12] Martagon AJ Zubirán R González-Arellanes R Praget-Bracamontes S Rivera-Alcántara JA Aguilar-Salinas CA. HDL abnormalities in type 2 diabetes: Clinical implications. Atherosclerosis (2023) 117213. doi: 10.1016/j.atherosclerosis.2023.117213 37580206

[13] Sacks FM Furtado JD Jensen MK. Protein-based HDL subspecies: Rationale and association with cardiovascular disease, diabetes, stroke, and dementia. Biochim Biophys Acta Mol Cell Biol Lipids (2022) 1867(9):159182. doi: 10.1016/j.bbalip.2022.159182 35605828

[14] Stadler JT van Poppel MNM Christoffersen C Hill D Wadsack C Simmons D. Gestational hypertension and high-density lipoprotein function: an explorative study in overweight/obese women of the DALI cohort. Antioxidants (Basel) (2022) 12(1):68. doi: 10.3390/antiox12010068 36670930 PMC9854490

[15] An R Ma S Zhang N Lin H Xiang T Chen M. AST-to-ALT ratio in the first trimester and the risk of gestational diabetes mellitus. Front Endocrinol (Lausanne) (2022) 13:1017448. doi: 10.3389/fendo.2022.1017448 36246899 PMC9558287

[16] Vujkovic M Ramdas S Lorenz KM Guo X Darlay R Cordell HJ. A multiancestry genome-wide association study of unexplained chronic ALT elevation as a proxy for nonalcoholic fatty liver disease with histological and radiological validation. Nat Genet (2022) 54(6):761–71. doi: 10.1038/s41588-022-01078-z PMC10024253 35654975

[17] Hanley AJ Wagenknecht LE Festa A D'Agostino RB Jr Haffner SM. Alanine aminotransferase and directly measured insulin sensitivity in a multiethnic cohort: the Insulin Resistance Atherosclerosis Study. Diabetes Care (2007) 30(7):1819–27. doi: 10.2337/dc07-0086 17429060

[18] Qian K Zhong S Xie K Yu D Yang R Gong DW. Hepatic ALT isoenzymes are elevated in gluconeogenic conditions including diabetes and suppressed by insulin at the protein level. Diabetes Metab Res Rev (2015) 31(6):562–71. doi: 10.1002/dmrr.2655 PMC4696510 25865565

[19] Ballestri S Zona S Targher G Romagnoli D Baldelli E Nascimbeni F. Nonalcoholic fatty liver disease is associated with an almost twofold increased risk of incident type 2 diabetes and metabolic syndrome. Evidence from a systematic review and meta-analysis. J Gastroenterol Hepatol (2016) 31(5):936–44. doi: 10.1111/jgh.13264 26667191

[20] Cao C Hu H Han Y Yuan S Zheng X Zhang X. The nonlinear correlation between alanine aminotransferase to high-density lipoprotein cholesterol ratio and the risk of diabetes: a historical Japanese cohort study. BMC Endocr Disord (2023) 23(1):124. doi: 10.1186/s12902-023-01382-7 37248447 PMC10226242

[21] Chen Y Zhang XP Yuan J Cai B Wang XL Wu XL. Data from: Association of body mass index and age with incident diabetes in Chinese adults: a population-based cohort study. (2018). doi: 10.5061/dryad.ft8750v PMC6169758 30269064

[22] Chen Y Zhang XP Yuan J Cai B Wang XL Wu XL. Association of body mass index and age with incident diabetes in Chinese adults: a population-based cohort study. BMJ Open (2018) 8(9):e021768. doi: 10.1136/bmjopen-2018-021768 PMC6169758 30269064

[23] American Diabetes Association. 2. Classification and diagnosis of diabetes: standards of medical care in diabetes-2018. Diabetes Care (2018) 41(Suppl 1):S13–27. doi: 10.2337/dc18-S002 29222373

[24] Zeng Q Li N Pan XF Chen L Pan A. Clinical management and treatment of obesity in China. Lancet Diabetes Endocrinol (2021) 9(6):393–405. doi: 10.1016/S2213-8587(21)00047-4 34022157

[25] Wax Y. Collinearity diagnosis for a relative risk regression analysis: an application to assessment of diet-cancer relationship in epidemiological studies. Stat Med (1992) 11(10):1273–87. doi: 10.1002/sim.4780111003 1518991

[26] Stefan N Cusi K. A global view of the interplay between non-alcoholic fatty liver disease and diabetes. Lancet Diabetes Endocrinol (2022) 10(4):284–96. doi: 10.1016/S2213-8587(22)00003-1 35183303

[27] Liu L Shao Y Li X Sun J Xing D. Individual and combined relationship of serum uric acid and alanine aminotransferase on metabolic syndrome in adults in Qingdao, China. Nutr Metab Cardiovasc Dis (2022) 32(12):2822–9. doi: 10.1016/j.numecd.2022.08.014 36180297

[28] Yang J Hou Y Zhang Q Wang Y. Normal serum alanine aminotransferase levels for screening metabolic dysfunction-associated fatty liver disease. J Formos Med Assoc (2023) 122(10):1092–3. doi: 10.1016/j.jfma.2023.05.013 37230915

[29] Simental-Mendía LE Rodríguez-Morán M Gómez-Díaz R Wacher NH Rodríguez-Hernández H Guerrero-Romero F. Insulin resistance is associated with elevated transaminases and low aspartate aminotransferase/alanine aminotransferase ratio in young adults with normal weight. Eur J Gastroenterol Hepatol (2017) 29(4):435–40. doi: 10.1097/MEG.0000000000000811 28009717

[30] Nguyen QM Srinivasan SR Xu JH Chen W Hassig S Rice J. Elevated liver function enzymes are related to the development of prediabetes and type 2 diabetes in younger adults: the Bogalusa Heart Study. Diabetes Care (2011) 34(12):2603–7. doi: 10.2337/dc11-0919 PMC3220830 21953798

[31] Li Y Wang J Han X Hu H Wang F Yu C. Serum alanine transaminase levels predict type 2 diabetes risk among a middle-aged and elderly Chinese population. Ann Hepatol (2019) 18(2):298–303. doi: 10.1016/j.aohep.2017.02.001 31040092

[32] Gao F Huang XL Jiang XP Xue M Li YL Lin XR. Independent effect of alanine transaminase on the incidence of type 2 diabetes mellitus, stratified by age and gender: A secondary analysis based on a large cohort study in China. Clin Chim Acta (2019) 495:54–9. doi: 10.1016/j.cca.2019.03.1636 30946812

[33] Wong CA Araneta MR Barrett-Connor E Alcaraz J Castañeda D Macera C. Probable NAFLD, by ALT levels, and diabetes among Filipino-American women. Diabetes Res Clin Pract (2008) 79(1):133–40. doi: 10.1016/j.diabres.2007.07.012 PMC4512638 17764776

[34] Leng J Zhang C Wang P Li N Li W Liu H. Plasma levels of alanine aminotransferase in the first trimester identify high risk chinese women for gestational diabetes. Sci Rep (2016) 6:27291. doi: 10.1038/srep27291 27264612 PMC4893691

[35] Fraser A Harris R Sattar N Ebrahim S Davey Smith G Lawlor DA. Alanine aminotransferase, gamma-glutamyltransferase, and incident diabetes: the British Women's Heart and Health Study and meta-analysis. Diabetes Care (2009) 32(4):741–50. doi: 10.2337/dc08-1870 PMC2660465 19131466

[36] Vozarova B Stefan N Lindsay RS Saremi A Pratley RE Bogardus C. High alanine aminotransferase is associated with decreased hepatic insulin sensitivity and predicts the development of type 2 diabetes. Diabetes (2002) 51(6):1889–95. doi: 10.2337/diabetes.51.6.1889 12031978

[37] De Silva NMG Borges MC Hingorani AD Engmann J Shah T Zhang X. Liver function and risk of type 2 diabetes: bidirectional mendelian randomization study. Diabetes (2019) 68(8):1681–91. doi: 10.2337/db18-1048 PMC7011195 31088856

[38] Eeg-Olofsson K Gudbjörnsdottir S Eliasson B Zethelius B Cederholm J NDR. The triglycerides-to-HDL-cholesterol ratio and cardiovascular disease risk in obese patients with type 2 diabetes: an observational study from the Swedish National Diabetes Register (NDR). Diabetes Res Clin Pract (2014) 106(1):136–44. doi: 10.1016/j.diabres.2014.07.010 25108897

[39] He Y Ronsein GE Tang C Jarvik GP Davidson WS Kothari V. Diabetes impairs cellular cholesterol efflux from ABCA1 to small HDL particles. Circ Res (2020) 127(9):1198–210. doi: 10.1161/CIRCRESAHA.120.317178 PMC7554159 32819213

[40] Yuan L Li-Gao R Verhoeven A van Eyk HJ Bizino MB Rensen PCN. Altered high-density lipoprotein composition is associated with risk for complications in type 2 diabetes mellitus in South Asian descendants: A cross-sectional, case-control study on lipoprotein subclass profiling. Diabetes Obes Metab (2023) 25(8):2374–87. doi: 10.1111/dom.15118 37202875

[41] Liu J Wang W Wang M Sun J Liu J Li Y. Impact of diabetes, high triglycerides and low HDL cholesterol on risk for ischemic cardiovascular disease varies by LDL cholesterol level: a 15-year follow-up of the Chinese Multi-provincial Cohort Study. Diabetes Res Clin Pract (2012) 96(2):217–24. doi: 10.1016/j.diabres.2011.12.018 22244364

[42] Fryirs MA Barter PJ Appavoo M Tuch BE Tabet F Heather AK. Effects of high-density lipoproteins on pancreatic beta-cell insulin secretion. Arterioscler Thromb Vasc Biol (2010) 30(8):1642–8. doi: 10.1161/ATVBAHA.110.207373 20466975

[43] Assmann G von Eckardstein A Funke H. High density lipoproteins, reverse transport of cholesterol, and coronary artery disease. Insights mutations Circ (1993) 87(4 Suppl):III28–34. 8462178

[44] Bray GA. Medical consequences of obesity. J Clin Endocrinol Metab (2004) 89(6):2583–9. doi: 10.1210/jc.2004-0535 15181027

[45] Bekkelund SI Jorde R. Alanine aminotransferase and body composition in obese men and women. Dis Markers (2019) 2019:1695874. doi: 10.1155/2019/1695874 31534560 PMC6732629

[46] Purcell M Flores YN Zhang ZF Denova-Gutiérrez E Salmeron J. Prevalence and predictors of alanine aminotransferase elevation among normal weight, overweight and obese youth in Mexico. J Dig Dis (2013) 14(9):491–9. doi: 10.1111/1751-2980.12072 23678860

[47] Davis CE Williams DH Oganov RG Tao SC Rywik SL Stein Y. Sex difference in high density lipoprotein cholesterol in six countries. Am J Epidemiol (1996) 143(11):1100–6. doi: 10.1093/oxfordjournals.aje.a008686 8633598

[48] Anagnostis P Stevenson JC Crook D Johnston DG Godsland IF. Effects of menopause, gender and age on lipids and high-density lipoprotein cholesterol subfractions. Maturitas (2015) 81(1):62–8. doi: 10.1016/j.maturitas.2015.02.262 25804951

[49] Mooradian AD Albert SG Haas MJ. Low serum high-density lipoprotein cholesterol in obese subjects with normal serum triglycerides: the role of insulin resistance and inflammatory cytokines. Diabetes Obes Metab (2007) 9(3):441–3. doi: 10.1111/j.1463-1326.2006.00636.x 17391174

[50] Kauma H Savolainen MJ Heikkilä R Rantala AO Lilja M Reunanen A. Sex difference in the regulation of plasma high density lipoprotein cholesterol by genetic and environmental factors. Hum Genet (1996) 97(2):156–62. doi: 10.1007/BF02265258 8566946

[51] Dong MH Bettencourt R Barrett-Connor E Loomba R. Alanine aminotransferase decreases with age: the Rancho Bernardo Study. PloS One (2010) 5(12):e14254. doi: 10.1371/journal.pone.0014254 21170382 PMC2999530

[52] Dong MH Bettencourt R Brenner DA Barrett-Connor E Loomba R. Serum levels of alanine aminotransferase decrease with age in longitudinal analysis. Clin Gastroenterol Hepatol (2012) 10(3):285–90.e1. doi: 10.1016/j.cgh.2011.10.014 22020064 PMC3288181

[53] Nakajima K Igata M Higuchi R Tanaka K Mizusawa K Nakamura T. Association of serum high-density lipoprotein cholesterol with high blood pressures at checkup: results of kanagawa investigation of total checkup data from the national database-9 (KITCHEN-9). J Clin Med (2021) 10(21):5118. doi: 10.3390/jcm10215118 34768637 PMC8584897

[54] Weijenberg MP Feskens EJ Kromhout D. Age-related changes in total and high-density-lipoprotein cholesterol in elderly Dutch men. Am J Public Health (1996) 86(6):798–803. doi: 10.2105/ajph.86.6.798 8659652 PMC1380397

[55] Barzilai N Atzmon G Schechter C Schaefer EJ Cupples AL Lipton R. Unique lipoprotein phenotype and genotype associated with exceptional longevity. JAMA (2003) 290(15):2030–40. doi: 10.1001/jama.290.15.2030 14559957

[56] Lee SH Park SY Choi CS. Insulin resistance: from mechanisms to therapeutic strategies. Diabetes Metab J (2022) 46(1):15–37. doi: 10.4093/dmj.2021.0280 34965646 PMC8831809

[57] Ye J. Mechanisms of insulin resistance in obesity. Front Med (2013) 7(1):14–24. doi: 10.1007/s11684-013-0262-6 23471659 PMC3936017

