# Supplementary material for: Alanine aminotransferase to high- density lipoprotein cholesterol ratio is positively correlated with the occurrence of diabetes in the Chinese population: a population-based cohort study
Source: Front Endocrinol (Lausanne). 2023 Nov 28;14:1266692. doi: 10.3389/fendo.2023.1266692 (PMC10715265; doi:10.3389/fendo.2023.1266692)
Supplement: Supplementary file 1 [file Table_1.docx]

Supplementary Table 1: Collinearity diagnostics steps.

|  | VIF | | | |
| --- | --- | --- | --- | --- |
|  | Step 1 | Step 2 | Step 3 | Step 4 |
| ALT/HDL-C ratio | 7 | 6.9 | 3 | 3 |
| Age | 1.4 | 1.4 | 1.4 | 1.4 |
| Sex | 3.2 | 3.1 | 3.1 | 3.1 |
| Height | 50.9 | 2.1 | 2.1 | 2.1 |
| Weight | 161.7 | NA | NA | NA |
| BMI | 95.3 | 1.5 | 1.5 | 1.5 |
| SBP | 2.2 | 2.2 | 2.2 | 2.2 |
| DBP | 2 | 2 | 2 | 2 |
| FPG | 1.1 | 1.1 | 1.1 | 1.1 |
| TC | 7 | 7 | 7 | NA |
| TG | 1.6 | 1.6 | 1.6 | 1.3 |
| HDL-C | 1.8 | 1.8 | 1.6 | 1.3 |
| LDL-C | 5.8 | 5.8 | 5.8 | 1.1 |
| ALT | 7.7 | 7.7 | NA | NA |
| AST | 3 | 3 | 2.6 | 2.6 |
| BUN | 1.2 | 1.2 | 1.2 | 1.2 |
| Cr | 2 | 2 | 2 | 2 |
| Family history of diabetes | 1 | 1 | 1 | 1 |
| Smoking status | 3.1 | 3.1 | 3.1 | 3.1 |
| Drinking status | 3.1 | 3.1 | 3.1 | 3.1 |

VIF: variance inflation factor;

VIF = 1/(1-R^2^). Abbreviations as in Table ​1.

Note: The variables with VIF>5 will be regarded as collinear variables and cannot be included in the multiple regression model
